# The Impact of Beta-Blocker Maintenance on Decompensated Heart Failure: A Systematic Review and Meta-Analysis

**DOI:** 10.2174/011573403X291307240902071924

**Published:** 2024-09-16

**Authors:** Luiz Fernando Leite da Silva Neto, Adriano Leitão de Almeida, Leticia Fonseca Macedo, Cauã Leal do Espírito Santo, Caio Vinicius Botelho Brito, Renato Garcia Lisboa Borges

**Affiliations:** 1 Biological and Health Sciences Center, Faculty of medicine, State University of Para, Belem, Brazil;; 2 Department of Community Health, Faculty of medicine, State University of Para, Belem, Brazil;; 3 Joao de Barros Barreto University Hospital, Federal university of Para, Belem, Brazil

**Keywords:** Heart failure, beta-adrenergic antagonists, mortality, hospitalization, systolic blood pressure, beta blockers

## Abstract

**Background:**

Acute Heart Failure (HF) is related to a significant hospital mortality rate and functional impairment in many patients. However, there is still a lack of studies that support the use of Beta-blockers (BB) in the management of decompensated HF. Objective: This study aimed to evaluate the impact on mortality of maintaining BB in patients with decompensated HF.

**Methods:**

A systematic review and meta-analysis was performed, using the databases PubMed, Cochrane Library, SCIELO and BVS, selecting only cohort studies and Randomized Clinical Trials (RCTs) from the last 10 years, which have been selected based on inclusion and exclusion criteria.

**Results:**

An 86% reduction in the risk of in-hospital death was found (RR=0.14, 95% CI: 0.10-0.18) in patients with HF who maintained the use of BB during hospitalization. A second analysis found a 44% (RR=0.56, 95% CI: 0.47-0.66) lower chance of in-hospital death in the group that previously used BB. Regarding the analysis of mortality after hospital discharge, only studies that have evaluated the use of BB in HF with reduced ejection fraction pointed to a reduction in mortality. Furthermore, some articles have found a relationship between the reduction in readmissions and the use of post-discharge BB.

**Conclusion:**

There is still no consensus regarding the use of BB in patients hospitalized with decompensated HF. In view of the limitations of the data found in the present study, the need for more RCTs that address this topic is emphasized in order to resolve this uncertainty in the management of cardiovascular patients.

## 1 INTRODUCTION

Acute Heart Failure (HF) represents a significant cause of hospitalization worldwide, particularly in individuals over the age of 65, being associated with high mortality rates and readmission rates [1]. Despite advances in the healthcare system regarding HF management, this disease remains a major challenge for physicians, with in-hospital mortality ranging from 4 to 10%, along with high 90-day readmission rates and 1-year mortality rates that can reach 10 to 30% [2-4].

Decompensated Heart Failure (DHF) represents a clinical syndrome defined by the heart's failure to eject and/or accommodate blood adequately to meet the body's metabolic needs, leading to functional limitation requiring emergency therapeutic intervention [5]. Although being one of the leading causes of hospitalization and associated with a high risk of mortality, especially in elderly patients, DHF still lacks sufficient evidence in the literature to support its proper management, particularly when compared to chronic heart failure [6, 7].

As one of the primary targets of DHF is the reduction of congestion, which can be present in 85% of patients, therapy involving loop diuretics forms the basis for managing these patients [1, 8]. Among these, furosemide stands out as the primary diuretic used. The clinical trial DOSE (Diuretic Optimization Strategies Evaluation) assessed the use of diuretics in HF patients, demonstrating high-dose furosemide as associated with symptom improvement, such as dyspnea and volume loss, despite being related to worsened renal function [6, 8, 9].

Furthermore, DHF encompasses another hemodynamic profile, characterized by congestion and inadequate perfusion of patients. In these patients, systemic blood pressure is an important guide for classifying and directing the therapy for Acute Heart Failure (AHF). Approximately 10% of patients present with Systolic Blood Pressure (SBP) < 90 mmHg. They may also exhibit inadequate peripheral perfusion, making them potential candidates for inotropic therapy and even vasopressors [10].

Through large randomized clinical trials, such as CIBIS-II, COPERNICUS, and MERIT-HF, the use of Beta-blockers (BBs) in chronic HF is well established in the literature [11]. However, in DHF, the use of this drug class is not yet well defined, as indicated by one of the recommendations in the 2021 European Guideline, which recommends their use only in hemodynamically stable patients [1]. Despite discrepancies in the literature regarding BB use in DHF, many studies have pointed to the benefits of their use in acute patients.

According to the joint guidelines of the American Heart Association (AHA), American College of Cardiology (ACC), and Heart Failure Society of America (HFSA), discontinuation of BBs was associated with an increased risk of mortality and rehospitalization [12]. Additionally, prior use of BBs in DHF patients reduced the incidence of cardiovascular events and stroke [13]. However, discontinuation of BBs should be considered in patients with significant volume overload or hypoperfusion, as well as in patients who require inotropes during hospitalization and were not using BBs previously [12].

However, the European Society of Cardiology has recently updated the AHF guideline, highlighting the STRONG-HF clinical trial, which has demonstrated the efficacy of early introduction of oral therapy for HF, including Angiotensin-converting Enzyme Inhibitors (ACEIs) or Angiotensin Receptor Blockers (ARBs), BBs, and mineralocorticoid receptor antagonists, around 2 days before hospital discharge. It is worth noting that the patients included in the study were hemodynamically stable [14, 15].

Even though in clinical practice, many physicians have discontinued BBs in patients with decompensated acute HF, justifying this decision by the potential worsening of hemodynamic stability, the lack of more concrete evidence to support this clinical decision remains a problem in the management of this cardiac emergency [11]. Therefore, this study has conducted a systematic review and meta-analysis with the objective of assessing the benefit for mortality when discontinuing or maintaining beta-blockers in patients with decompensated heart failure, in addition to analyzing the impact on mortality of the prior use of beta-blockers upon admission and evaluating the effect of beta-blockers on the rehospitalization of these patients.

## 2 METHODS

### 2.1 Protocol

This study involved a systematic review, followed by a meta-analysis, employing a quantitative approach, based on the recommendations of the Preferred Reporting Items for Systematic Reviews and Meta-Analyses (PRISMA). To conduct the research, a guiding question was developed based on the acronym “PICO,” as follows: P (population); I (intervention); C (comparison); O (outcome). Therefore, the central research question was “Is there an impact on mortality following the maintenance of beta-blockers in patients with decompensated heart failure?”, with each item of PICO presented in Table **1**.

### 2.2 Ethical Considerations

The research was conducted following the principles established in the Nuremberg Code and the Declaration of Helsinki. It was not necessary to submit to an ethics committee as it was a systematic literature review, followed by a meta-analysis that did not directly involve human subjects.

### 2.3 Inclusion and Exclusion Criteria

Studies that included patients hospitalized with HF were eligible. Additionally, studies characterized as randomized clinical trials and cohort studies were included, provided that they have been published, indexed with the used descriptors, and freely available in their entirety in the following languages: Portuguese, English, and Spanish. Furthermore, the studies suitable for the research must have been published in the last 10 years, from 2013 to 2023. It is worth noting that this extended time frame has been due to the COVID-19 pandemic, which has delayed research in this field.

On the other hand, repeated studies, those that have shown increased mortality due to secondary causes from the discontinuation of beta-blockers, or studies involving a study population under 18 years of age have been excluded.

### 2.4 Search Strategy

The search for articles was conducted in the following databases: PubMed, Cochrane Library, Scientific Electronic Library Online (SCIELO), and the Virtual Health Library (BVS) during the month of April 2023.

Furthermore, the descriptors were collected from the Health Sciences Descriptors (HSD), also using the Medical Subject Headings (MeSH) system. Consequently, a cross-reference was made with Boolean operators “AND” and “OR,” obtaining the relationship presented in Table **2**.

### 2.5 Study Selection

Following the database search, studies were selected using the Rayyan platform, based on independent peer evaluation by comparing study information with the eligibility criteria established in this study. In case of disagreement regarding a particular study, a third evaluator was involved to finalize the decision. The study selection went through three stages: removal of duplicates, title and abstract reading, and finally, full-text article reading, with the process based on PRISMA.

### 2.6 Data Collection and Bias Analysis

The selected articles were included in the review to perform the results. The main information from each study was extracted by two independent reviewers, with the possibility of a third reviewer in case of conflicting information. Data were stored and tabulated in the Excel 365 platform, with a summary of the studies presented in Table **3**, along with their authors, publication year, study type, population characteristics, sample size, the number of patients using beta-blockers, gender, average age, outcomes analyzed, and main conclusions.

Additionally, the following outcomes were extracted to complement the analysis of results: the number of in-hospital deaths and follow-up deaths as well as the number of rehospitalizations.

The quality of the studies was independently evaluated by 3 reviewers using the Rob 2.0 tool [16] for randomized clinical trials, analyzing the risk of bias in randomized clinical trials in domains, including the randomization process, deviation from the intended intervention, lack of outcome data, outcome measurement, and selection of reported outcomes. Afterward, articles were assessed for each domain, indicating “low risk of bias,” “some concerns,” or “high risk of bias.” Finally, cohort studies included in the study were assessed using the Newcastle-Ottawa Scale [17], composed of 3 domains scoring methodological quality: selection, with up to 4 points; comparability, with up to 2 points; outcome, with up to 3 points.

### 2.7 Meta-analysis

The included studies were presented in a forest plot, analyzing the risk ratio of the intervention (maintenance of beta-blockers) compared to the control (discontinuation of beta-blockers) for outcomes. Additionally, secondary analyses were conducted, stratifying the study based on rehospitalization and prior use of beta-blockers upon admission.

For this purpose, the meta-analysis was conducted using the Review Manager Web program, employing a fixed-effects model for homogeneous study sets (i < 0.5) and a random-effects model for heterogeneous study sets (i ≥ 0.5), considering a 95% confidence interval. In the cases of high heterogeneity, subgroup analysis was performed, excluding studies responsible for the discrepancy if possible.

## 3 RESULTS

**Fig. (1):**
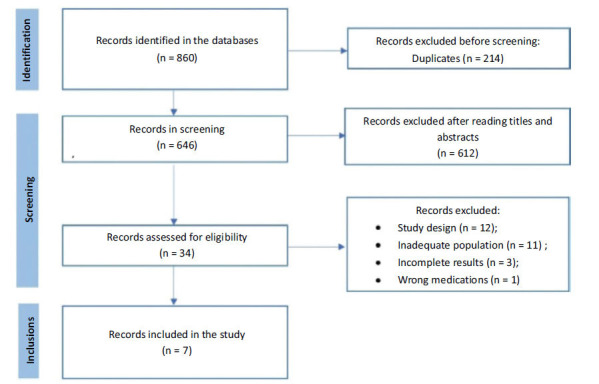
PRISMA Flowchart of the article selection process.

In total, 860 articles were identified, with 214 excluded as duplicates, resulting in 646. After reading the titles and abstracts, 612 studies were removed as they were not compatible with the research, leaving 34 articles for full evaluation. Out of these, 12 were excluded due to an inappropriate study design, 11 had an inadequate population, 3 had incomplete results, and 1 did not feature the use of beta-blockers. As a result, 7 studies were included in the final selection (Fig. **1**). Furthermore, the selected articles have been summarized and presented in Table **3**.

### 3.1 Bias Analysis

In the evaluation of the included randomized clinical trial [18], a moderate risk of bias was found.

Regarding the bias domain related to the randomization process, a low risk was observed because the sequence was concealed, followed by an appropriate randomization process, and no significant demographic differences between the two groups.

In terms of deviations from intended interventions, the study was classified as having a moderate risk of bias because there was awareness of the intervention.

Concerning bias due to loss of follow-up, the study had a low risk because data loss was not significant, or any loss was properly corrected or balanced between the groups.

Regarding outcome measurement, the study had a low risk of bias because the results were measured in a way that could not be influenced by knowledge of the intervention received by the treatment group.

**Fig. (2):**
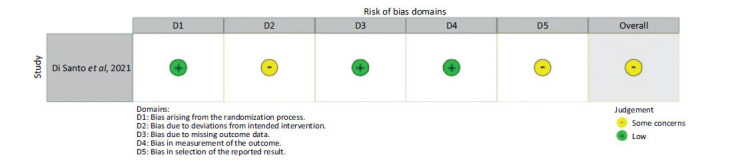
Bias assessment of the included randomized clinical trial in the meta-analysis, according to ROB 2.0.

As for the selection of the reported outcome, a moderate risk of bias was found because it involved a subgroup analysis from a larger cohort, and such an analysis was not described in the initial study protocol (Fig. **2**).

In the evaluation of observational studies, shown on Table **4** three out of the six studies achieved the maximum methodological quality score. Among the others, only Wu and collaborators (2018) [23] lost one star in the selection category due to the absence of the outcome (rehospitalization) at the beginning of the follow-up. Two studies did not score in comparability [13, 23] due to the absence of clinical description of the comparison groups. Finally, two studies lost one point in the outcome assessment [19, 23], both for not describing study follow-up losses.

### 3.2 Meta-analysis

**Fig. (3):**
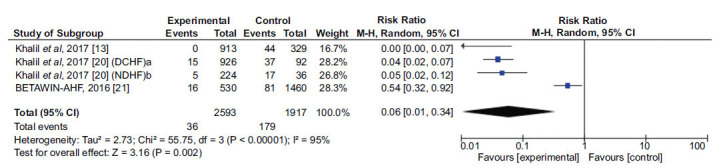
Analysis of the effect of the maintenance of beta-blockers on in-hospital mortality in patients with decompensated heart failure. ^a^DCHF: Decompensated chronic heart failure. ^b^NDHF: Newly diagnosed heart failure.

Three studies [13, 20, 21] were included in the statistical analysis for comparing the maintenance of Beta-blocker (BB) use, with a total of 2593 patients in the sample, to the suspension of this medication, with 1917 participants. The study by Khalil and collaborators (2017) [20] analyzed two distinct cohorts, one with patients with decompensated Chronic Heart Failure (CHF) and another with patients recently diagnosed with CHF. The analyzed outcome was all-cause in-hospital mortality. It was observed that the group that continued medication had a 94% lower chance of developing the outcome (RR=0.06, 95% CI: 0.01-0.32) (Fig. **3**).

The analysis showed a 95% heterogeneity, with no significant reduction after the removal of any study, so a random-effects analysis was performed. Khalil and collaborators (2017) [20], who presented the most significant indicators, included 1278 patients with reduced Ejection Fraction (EF) and prior use of BB upon admission, and separately analyzed the effects of discontinuing the class of medication in patients with decompensated chronic heart failure and recently diagnosed acute heart failure. However, the study did not describe the clinical characteristics of the comparison groups in both populations and indicated independent risk factors for mortality, such as the need for inotropic agents, without also describing if these factors have been different between maintenance and discontinuation. Nevertheless, the study indicated blood pressure, adherence to drug therapy, and the use of inotropic agents to be different between the groups and considered for statistical adjustment, after which the odds ratio remained significantly reduced for mortality with the maintenance of BB in patients with decompensated chronic heart failure (OR=0.084, 95% CI: 0.015-0.468) and recent acute heart failure (OR=0.047, 95% CI: 0.013-0.169).

Similarly, Khalil and collaborators (2017) [13] evaluated patients admitted with Decompensated Heart Failure (DHF) regarding prior BB use upon admission, conducting a secondary analysis with 1242 patients using the medication to assess the effects of its discontinuation. They also found a considerable benefit of maintaining the class in in-hospital mortality, with no deaths in the maintenance group. Again, the clinical characteristics of the groups were not described, although it was reported that the use of inotropic agents and cardiovascular events during hospitalization were similar between the groups.

Finally, BETAWIN-AHF [21] evaluated data from 1990 patients admitted to hospitals in Spain, separating cohorts of in-hospital BB suspension versus maintenance. The maintenance group was younger, had a higher frequency of atrial fibrillation, greater use of aldosterone antagonists, a higher Heart Rate (HR) at admission, and lower Systolic Blood Pressure (SBP) than the suspension group, with similarities in other factors, such as comorbidities, functionality, ejection fraction, and clinical parameters. Based on this, the study found a slight increase in in-hospital mortality with BB suspension, even after adjusting for confounding factors (OR 1.77, 95% CI: 1.01–3.09), with an additional effect in patients with HR > 80 bpm at admission (OR 2.74; 95% CI: 1.13–6.63). The protective effect of BB was preserved in the analysis based on the relative risk (RR 0.54; 95% CI: 0.32–0.92), indicating an average reduction of 46% in in-hospital mortality, with a drug-attributable risk of -3.32% and a number needed to treat of 30.16.

**Fig. (4):**
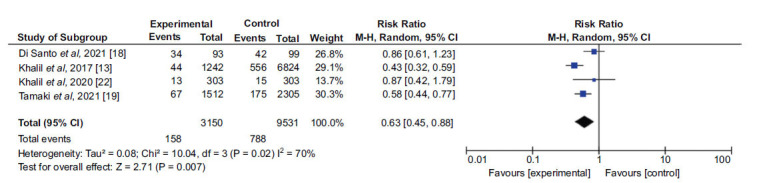
Analysis of the effect of prior beta-blocker use on in-hospital mortality in patients with decompensated heart failure.

**Fig. (5):**
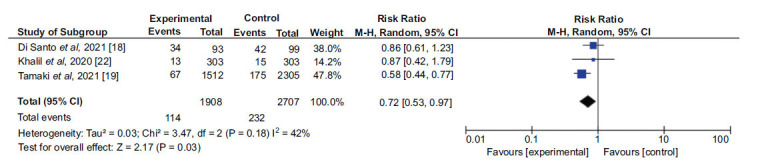
Analysis of the effect of prior beta-blocker use on in-hospital mortality in patients with decompensated heart failure, after controlling for study heterogeneity.

In addition, 4 studies [13, 18, 19, 22] were included in a second analysis to evaluate the outcome by comparing two groups: patients admitted with prior beta-blocker use and patients admitted without prior use. The first sample consisted of 3150 patients, while the second sample consisted of 9531. A 37% lower chance of the outcome occurring was found in the group using the medication. There was a 70% heterogeneity when analyzing the 4 studies, and a random-effects analysis was also performed. Due to the high heterogeneity found, an analysis was performed by excluding the article by Khalil and collaborators (2017) [13], resulting in a heterogeneity of 42%, while still maintaining a protective effect of prior BB use (Figs. **4** and **5**).

With respect to the relationship between post-discharge mortality and the decision made regarding the withdrawal or use of BB in ACD, three studies have addressed this issue in patients with reduced ejection fraction from a different temporal perspective. Wu and collaborators (2018) [23] associated the continued use of BB with lower mortality after 1-year post-discharge, accompanied by a reduction in cardiovascular events (RR = 0.71, 95% CI: 0.61-0.82). Miró and collaborators (2016) [21] obtained the same result in 30 days of follow-up (RR = 1.93, 95% CI: 1.22-3.06). Khalil and collaborators (2017) [20] highlighted the impact on reduced mortality at 3 months (RR = 0.51, 95% CI: 0.23-1.14). However, there was a fourth study that addressed the same context, but in patients with preserved ejection fraction; Khalil and collaborators (2020) [22] reported a lack of beneficial results in mortality over 12 months of follow-up (RR = 0.86, 95% CI: 0.51-1.45).

Regarding the variable of re-hospitalization, two studies indicated a weak association between BB use or discontinuation (during hospitalization and after discharge) and the emergency department rehospitalization rate (RR = 1.17, 95% CI: 0.92–1.48) [20,21]. Ainda, Khalil, and collaborators (2020) [22] found BB use at discharge to not be associated with a reduction in hospitalization for heart failure (RR = 0.95; 95% CI: 0.64-1.40, *p* = 0.80).

However, in Kalil and collaborators' research study (2018) [23], 11,012 patients diagnosed with ACD during hospitalization were analyzed, and an association was found between BB use and a reduction in heart failure rehospitalization from 30 days up to 12 months (30 days: RR = 0.60, 95% CI = 0.37–0.97; 180 days: RR = 0.76, 95% CI = 0.60–0.98; 365 days: RR = 0.72, 95% CI = 0.58–0.90). The only exception was that this benefit was not significant in the 14-day readmission (RR = 0.69; *p* = 0.273).

## 4 DISCUSSION

The studies have included a total of 20,137 participants, of which 4,491 were on beta-blockers. The average age was 69.8 years (ranging from 59 to 81), with 53.97% being male. Regarding the presence of comorbidities, the majority reported having hypertension, diabetes mellitus, obesity, stroke, coronary artery disease, prior percutaneous intervention or coronary artery bypass surgery, and atrial fibrillation.

Results for the primary objective indicated an average 94% reduction in the risk of in-hospital death with beta-blocker maintenance, with an attributable risk of 7,95%. Initially, a significant benefit was found for beta-blocker maintenance during hospitalization for acute decompensated heart failure compared to discontinuation. However, this effect was associated with a high level of heterogeneity among the studies and abnormally significant relative risk results, reducing the reliability of the effect found.

Initially, the fact that the studies were observational has hampered the assessment of the true effect of the intervention because the choice between maintenance and discontinuation of medications was made based on clinical criteria rather than randomization. In this context, several factors have influenced this decision. Beta-blocker maintenance might be favored in populations with associated comorbidities where the medication is part of the therapy, such as atrial fibrillation. On the other hand, discontinuation of the drug could be influenced by medication intolerance in frail or elderly patients or due to limited evidence regarding this class of drugs in patients with preserved ejection fraction during the study period, reducing maintenance in these groups. Furthermore, healthcare providers may systematically discontinue beta-blockers in patients with decompensated heart failure, justifying the need to stabilize the patient's condition before resuming their use. The same logic can be applied to individualized decisions, suspending the drug in patients with hemodynamic instability who require vasoactive drugs and inotropes [13, 21].

Some of these factors theoretically have a neutral effect or favor the discontinuation group, while others may affect the results in favor of the maintenance group, such as patient frailty and, most importantly, hemodynamic instability, influencing the decision to discontinue beta-blockers in the studies, creating discontinuation groups that are clinically more severe than the maintenance groups.

In this assessment, Khalil and collaborators (2017) [13], [20] have failed to provide clinical characteristics between the comparison groups, with limited information on the severity and frailty profile of the patients, making it difficult for the reader to analyze these possible confounding factors or whether such factors were even evaluated by the studies. As a result, the lack of transparency in the data from these studies, combined with the persistence of the unrealistic effect of beta-blocker maintenance even after adjusting for variables, has indicated a considerable probability of selection bias and/or confounding.

On the other hand, BETAWIN-AHF [21] was the best study in terms of transparency and methodological quality, evaluating the discontinuation versus maintenance of beta-blockers. It demonstrated good comparability between the groups and effective control of confounding factors, in contrast to the other research studies, which have found significant intervention effects at the cost of low methodological quality. Therefore, the real effect of maintaining beta-blockers in heart failure hospitalizations appears to be a modest benefit, with more advantages in specific populations, such as patients with a high heart rate or those with associated conditions, like atrial fibrillation. However, a more precise measurement of this effect could require randomization.

Despite advances in chronic heart failure treatment in recent years, acute heart failure still maintains a high rate of in-hospital mortality. In addition to the high mortality, each hospitalization for acute heart failure further increases the risk of rehospitalization, leading to cardiac dysfunction and worsening prognosis for these patients. Therefore, post-discharge follow-up and the implementation of evidence-based therapies, such as beta-blockers, ACE inhibitors/ARBs, and spironolactone, can reduce mortality and readmission rates, while improving long-term survival [24, 25].

Among the factors related to the low statistical significance between the use of beta-blockers during hospitalization and the risk of rehospitalization over 3 to 6 months, patient adherence to medication at home, social and family support, and the use of this medication in individuals with preserved left ventricular ejection fraction stand out [20, 21]. This is because the use of beta-blockers in patients with reduced ejection fraction is well-established in the literature (class I, level A) [12,14,15]. Furthermore, beta-blockers are associated with the pathophysiological mechanism of Heart Failure with reduced Ejection Fraction (HFrEF), as the medication protects against sympathetic system overactivity and the increase in catecholamines related to systolic dysfunction. However, sympathetic system overactivity does not appear to be responsible for diastolic dysfunction [26].

Studies have shown that a low heart rate is associated with a better prognosis in heart failure patients treated with beta-blockers, while a heart rate greater than 70 bpm is related to worsened heart failure or mortality [27]. Moreover, in hospitalized patients, maintaining a low heart rate is also associated with a lower incidence of cardiac events [28]. Therefore, it is believed that there is a benefit to using beta-blockers during hospitalization for acute heart failure, especially when combined with other medications [29]. A meta-analysis conducted by Prins and collaborators (2015) [11] stated discontinuing the use of beta-blockers as associated with a significant increase in mortality and rehospitalization in patients with decompensated heart failure. However, the use of beta-blockers in these patients is still not assured, despite the results found in the meta-analysis conducted in this study, because although the survival values have been significant, the studies analyzed had limitations.

The results showed a reduction in mortality among patients with reduced ejection fraction (HFrEF) who were previously using beta-blockers upon hospital admission. This outcome may be related to the mechanism of this HFrEF profile, characterized by the action of the sympathetic nervous system, the presence of catecholamines, and the stimulation of cardiac beta-adrenergic receptors, which promote inotropic and chronotropic effects, *i.e*., increasing ventricular contractility and heart rate, respectively. With the use of beta-blockers, the pathophysiological cascade of HFrEF is minimized, demonstrating survival benefits [30, 31]. On the other hand, when evaluating patients with Heart Failure with preserved Ejection Fraction (HFpEF), this benefit from beta-blockers is not observed. The study by Silverman and collaborators (2019) [32], a secondary analysis of a randomized clinical trial in patients with EF > 50%, indicated that these patients, when using beta-blockers, have a higher risk of hospitalization. Furthermore, in hospitalized HFpEF patients, previous drug therapy did not show benefits on mortality, as its impact on survival was not relevant [22].

However, the reasons why HFpEF patients do not benefit from pre-hospitalization drug therapy, given that its impact on survival is almost negligible, remain unknown [22].

In addition to evaluating the use of beta-blockers during hospitalization, the present study has also assessed the use of beta-blockers before hospitalization. The study by Khalil and collaborators (2020) [22] demonstrated no significant difference in the occurrence of adverse vascular events and mortality between patients using beta-blockers and those not using them. However, the study by Tamaki and collaborators (2021) [19] pointed out a significant reduction in mortality among patients treated with beta-blockers. Similarly, another study showed fewer deaths in the group of patients using this class of medications, but without differences in other analyzed outcomes, indicating the benefit as related to a reduction in early events and having no observable benefit for the rest of the hospitalization period [18]. In summary, these studies suggest an uncertain benefit of pre-hospitalization beta-blocker use when assessing the outcome of mortality.

The main limitation of the study relates to the low number of studies addressing this topic, especially randomized clinical trials, which may be associated with the difficulty of conducting studies with this methodological characteristic in hemodynamically unstable patients, such as in decompensated HF, leading to the difficulty in implementing randomized-based approaches since decision-making is usually immediate in patient care. Therefore, observational studies on the topic are commonly found in the literature.

Furthermore, there was a lack of a homogeneous profile of the analyzed population, resulting from the methodological criteria proposed by the studies selected for review, leading to heterogeneous results. Part of the included studies have also failed to report relevant clinical characteristics of the patients, lacking a well-detailed subgroup evaluation and making less transparent the variables-adjusted analysis, which may have influenced the results of studies with low methodological quality. Researchers who took these precautions, even if observational, found relevant answers that have guided the discussion of this study and can also guide the design of future studies.

## CONCLUSION

Regarding the previous use of beta-blockers upon hospital admission for patients with decompensated HF, this meta-analysis has found a reduction in mortality among patients with reduced ejection fraction. Additionally, for individuals with preserved ejection fraction, the impact of beta-blockers had no effect on their prognosis. As for re-hospitalization, the use of this class of medication showed beneficial effects, mainly due to the relationship between heart rate reduction and a promising outcome.

In conclusion, the use of beta-blockers during a decompensated HF episode may result in a substantial reduction in in-hospital mortality. However, despite this outcome being consistent with the literature, this benefit may be subject to distortion due to high heterogeneity, caused by methodological flaws in the studies and the scarcity of randomized clinical trials addressing this type of intervention in this specific study group. Therefore, the use of beta-blockers should be individualized in different populations within different clinical contexts, depending on their clinical and hemodynamic status requiring further studies with these specific characteristics. Despite the limitations encountered, this review has gathered evidence reinforcing the recommendations of recent guidelines and directing future research with greater rigorous methods to obtain a more reliable assessment of the real effect of maintaining or discontinuing beta-blockers in patients with decompensated HF.

## Figures and Tables

**Table 1:** Characterization of the guiding research question.

**Components**	**Description**
Population	Patients with decompensated heart failure
Intervention	Use of beta-blocker
Comparison	Discontinuation of beta-blocker
Outcome	Mortality

**Table 2:** Search strategies in the databases.

**Databases**	**Search Strategies**
Pubmed	(“heart failure” OR “cardiogenic shock” OR “heart failure acute” OR “acute heart failure” OR “acute heart failure decompensation”) AND (“beta blocker” OR “beta blocker agent” OR “beta blocker agents” OR “beta blocker antihypertensive” OR “adrenergic beta 2 receptor antagonists” OR “adrenergic beta antagonists”) AND (“mortality” OR “death” OR “hospitalization”).
SCIELO	(“Insuficiência Cardíaca” OR “choque cardiogênico” OR “cardiogenic shock” OR “Heart failure” OR “insuficiência cardíaca aguda” OR “insuficiência cardíaca descompensada” OR “Insuficiencia Cardíaca” OR “acute heart failure” OR “heart failure acute”) AND (“Antagonistas Adrenérgicos beta” OR “Adrenergic beta-Antagonists” OR “Antagonistas Adrenérgicos beta” OR “beta blocker” OR “Beta blockers” OR “betabloqueador” OR “beta bloqueador” OR “beta-bloqueador”) AND (mortalidade OR morte OR óbito OR mortes OR óbitos OR mortality OR death OR deaths OR Muerte OR hospitalizações OR hospitalizations OR morbidade).
Cochrane	(“heart failure” OR “cardiogenic shock”) AND (acute OR descompensated OR descompensation OR worsening) (“beta-Blockers, Adrenergic” OR “beta Adrenergic Antagonist” OR “beta-blocker”) (mortality OR morbity OR hospitalization) NOT chronic.
BVS	(“heart failure” OR “cardiogenic shock” OR “insuficiência cardíaca” OR “insuficiencia cardíaca” OR “choque cardiogênico” OR “Insuficiência cardíaca aguda” OR “Insuficiência cardíaca descompensada” OR “Acute heart failure”) and (betabloqueador OR “Adrenergic beta-Antagonists” OR “Antagonistas Adrenérgicos beta” OR “beta blockers”) and (death OR mortality OR hospitalization OR óbitos OR mortes OR muertes OR hospitalização OR internação OR hospitalizações OR internações OR hospitalizados OR internados OR hospitalización OR mortalidade OR mortalidad).

**Table 3:** General characteristics of the studies included in the Systematic Review.

**Authors and Year**	**Study Design**	**Population**	**Number of Participants (and in Use of BB)**	**Proportion of men (%)**	**Mean age**	**Analyzed Outcomes**	**Conclusion**
Di Santo *et al.* (2021) [18]	RCT	Patients with cardiogenic shock using Milrinone or Dobutamine.	192 (93)	122 (63,5%)	70,7 years	In-hospital mortality and cardiovascular events.	Lower mortality and need for cardiopulmonary resuscitation in the group previously using BB.
Tamaki *et al.* (2021) [19]	Prospective cohort	Patients at first episode of decompensated HF.	3817 (1512)	2086 (54,6%)	81 years	All-cause and per etiology in-hospital mortality.	Lower mortality in the group previously using BB.
Khalil *et al.* (2017) [20]	Prospective cohort	Patients hospitalized with decompensated HF of EF<40% previously using BB.	1278 (1140)	928 (72,61%)	59 years	In-hospital mortality, at 3 and 12 months, length of stay, Rehospitalization at 3 and 12 months.	Lower in-hospital mortality with BB maintenance, with no difference in length of stay or follow-up outcomes.
Miró *et al.* (2016) (BETAWIN-AHF) [21]	Prospective cohort	Patients hospitalized with decompensated HF previously using BB.	1990 (530)	887 (44,57%)	78,1 years	In-hospital all-cause mortality and at 30 days, length of stay, return to the emergency department.	Lower in-hospital and 30-day mortality with BB maintenance, with no difference in other outcomes.
Khalil *et al.* (2020) [22]	Prospective cohort	Patients hospitalized with decompensated HF of EF>40% and a history of coronary disease.	606(303)	322 (53,13%)	65 years	In-hospital mortality, cerebrovascular events, and cardiogenic shock.	No difference between the groups in the outcomes assessed.
Wu *et al.* (2018) [23]	Prospective cohort	Patients hospitalized with decompensated HF.	11012 (No information)	5680 (51,8%)	71,6 years	In-hospital mortality, cerebrovascular events, and rehospitalization within 1 year.	Lower in-hospital mortality and cardiovascular events and reduced rehospitalization at 1 year with prior use of BB.
Khalil *et al.* (2017) [13]	Retrospective cohort	Patients hospitalized with decompensated HF.	1242 (913)	844 (68%)	63,7 years	In-hospital mortality, length of stay, and cardiovascular events.	Lower in-hospital mortality and cardiovascular events with BB maintenance.

**Note:** Legend: RCT: Randomized clinical trial; HF: Heart failure; BB: Betablocker; EF: Ejection fraction.

**Table 4:** The methodological quality of observational studies included in the meta-analysis according to the Newcastle-Ottawa Scale.

**Study**	**Selection**	**Comparability**	**Outcome**	**Total**
Khalil *et al.*, 2017^13^	★★★★	☆☆	★★★	7/9 stars
Tamaki *et al.*, 2021^19^	★★★★	★★	★★☆	8/9 stars
Khalil *et al.*, 2017^20^	★★★★	★☆	★★★	8/9 stars
BETAWIN-AHF, 2016^21^	★★★★	★★	★★★	9/9 stars
Khalil *et al.*, 2020^22^	★★★★	★★	★★★	9/9 stars
Wu *et al.*, 2018^23^	★★★☆	☆☆	★★☆	5/9 stars

## Data Availability

All the data and supportive information are available within the article.

## LIST OF ABBREVIATIONS

ACC: American College of Cardiology
ACEi: Angiotensin-Converting Enzyme Inhibitors
AHA: American Heart Association
ARB: Angiotensin Receptor Blockers
BB: Beta-blockers
DCHF: Decompensated Chronic Heart Failure
DHF: Decompensated Heart Failure
EF: Ejection Fraction
HF: Heart Failure
HFpEF: Heart Failure with Preserved Ejection Fraction
HErEF: Heart Failure with Reduced Ejection Fraction
HFSA: Heart Failure Society of America
HSD: Health Sciences Descriptors
HT: Heart Rate
MeSH: Medical Subject Headings
NDHF: Newly Diagnosed Heart Failure
PRISMA: Preferred Reporting Items for Systematic Reviews and Meta-analyses
SBP: Systolic Blood Pressure
SCIELO: Scientific Eletronic Library Online

## SUPPLEMENTARY MATERIAL

PRISMA checklist is available as supplementary material on the publisher’s website along with the published article.
